# Effects of Aromatherapy on the Anxiety, Vital Signs, and Sleep Quality of Percutaneous Coronary Intervention Patients in Intensive Care Units

**DOI:** 10.1155/2013/381381

**Published:** 2013-02-17

**Authors:** Mi-Yeon Cho, Eun Sil Min, Myung-Haeng Hur, Myeong Soo Lee

**Affiliations:** ^1^Eulji University Hospital, Daejeon 302-799, Republic of Korea; ^2^College of Nursing, Eulji University, Daejeon 302-832, Republic of Korea; ^3^Medical Research Division, Korea Institute of Oriental Medicine, Daejeon 305-811, Republic of Korea

## Abstract

The purpose of this study was to investigate the effects of aromatherapy on the anxiety, sleep, and blood pressure (BP) of percutaneous coronary intervention (PCI) patients in an intensive care unit (ICU). Fifty-six patients with PCI in ICU were evenly allocated to either the aromatherapy or conventional nursing care. Aromatherapy essential oils were blended with lavender, roman chamomile, and neroli with a 6 : 2 : 0.5 ratio. Participants received 10 times treatment before PCI, and the same essential oils were inhaled another 10 times after PCI. Outcome measures patients' state anxiety, sleeping quality, and BP. An aromatherapy group showed significantly low anxiety (*t* = 5.99, *P* < .001) and improving sleep quality (*t* = −3.65, *P* = .001) compared with conventional nursing intervention. The systolic BP of both groups did not show a significant difference by time or in a group-by-time interaction; however, a significant difference was observed between groups (*F* = 4.63, *P* = .036). The diastolic BP did not show any significant difference by time or by a group-by-time interaction; however, a significant difference was observed between groups (*F* = 6.93, *P* = .011). In conclusion, the aromatherapy effectively reduced the anxiety levels and increased the sleep quality of PCI patients admitted to the ICU. Aromatherapy may be used as an independent nursing intervention for reducing the anxiety levels and improving the sleep quality of PCI patients.

## 1. Introduction 

Coronary artery diseases are ischemic heart diseases in which the coronary arteries narrow or close, which decrease the oxygen supply to the cardiac muscle, leading to various clinical symptoms such as angina pectoris, myocardial infarction, congestive heart failure, and sudden death. The prevalence of coronary artery diseases have increased recently as the population ages and as changes in eating habits and lifestyle have led to higher incidences of diabetes, high blood pressure, and hyperlipidaemia in Korea. Cardiovascular diseases ranked as the third leading cause of death and are among causes of circulatory system mortality; cardiac diseases rank second after neurovascular diseases in 2008. The mortality rate of ischemic heart disease in 100,000 people increased from 16.2% in 1998 to 25.7% out of 100,000 as mortality in 2008 [1]. For percutaneous coronary intervention (PCI), the standard procedure of coronary angiography is commonly used [2]. Percutaneous transluminal angioplasty and stent insertion using coronary angiography are important procedures for determining the location and severity of the blocked artery, and they can play an important role in determining subsequent treatment methods [3].

Coronary artery diseases such as myocardial infarction and angina pectoris generally lead to hospitalisation in an intensive care unit (ICU). Most of these patients experience the physical effects of a heart examination as well as an unfamiliar environment, isolation from family, and stress from encountering strangers. Consequently, most patients experience a relatively severe level of psychological anxiety because of the loss of individuality due to the treatment-centric environment and a sense of crisis due to the constantly changing environment [4]. Additionally, current ICUs have state-of-the-art patient surveillance, treatment instruments and apparatuses, and sudden emergency situations can occur at any time. Environmental factors including excessive noise, constant light, unpleasant odours, frequent care, crisis situations, and fear of diseases interfere with sleep [5]. The environmental stress and unresolved anxiety interfere with patient security, care, and sleep. Anxiety, stress, and insomnia significantly affect the treatment of the coronary artery disease, which can lead to an increase in the area of infarction and even to arrhythmia [6]. Therefore, independent nursing interventions to decrease anxiety and stress and increase the sleep quality of ICU patients with coronary artery diseases are necessary.

Because nursing interventions based on education before coronary angiography have shown to be effective, structured care and education by providing information before the procedure have been implemented in the clinical setting. Aromatherapy, which has a wide range of applications and is easy to deploy, has recently garnered much attention [7]. Specifically, efforts to scientifically demonstrate the effects of aromatherapy as a holistic intervention and as a relaxation mediator have been actively pursued in nursing [8]. Aromatherapy has been reported to reduce stress [9, 10] as well as decrease anxiety and increase sleep quality in cancer patients [11], haemodialysis patients [12], and colonoscopy patients [13]. Aromatherapy is noninvasive and can be applied continuously to patients who do not have an aversion to the odours. Therefore, the effects of aromatherapy on anxiety, stress, and short-term sleep in subjects undergoing coronary angiography need to be assessed. Same-day aromatherapy has shown to be effective in decreasing preprocedure anxiety and to increase sleep in inpatients undergoing cardiac angiography [14]. However, there was no study of effects of aromatherapy on the postprocedure disturbances of patients undergoing stent insertion during coronary angiography. Therefore, we tested the effect of aromatherapy on the anxiety, blood pressure, and sleep of patients with ischemic heart diseases who underwent stent insertion during coronary angiography and were admitted to a cardiovascular ICU.

## 2. Participants and Methods

### 2.1. Study Design

This study is a nonequivalent control group nonsynchronised quasiexperiment designed to assess the effect of a lavender, roman chamomile, and neroli oil blend aromatherapy on anxiety, sleep, and blood pressure in coronary artery disease patients with ischemic heart diseases after a stent insertion during coronary angiography who were admitted to the ICU. 

### 2.2. Participants

The subjects of this study were patients diagnosed with coronary artery diseases who underwent a stent insertion during coronary angiography and were admitted to the cardiovascular ICU between August 1, 2010, and November 20, 2010, at the Eulji University Hospital in Daejeon, Korea. This study was approved by the Institutional Review Board at Eulji University before data collection (EU 10–14). The specific criteria for subject selection were patients diagnosed with angina pectoris or myocardial infarction who had coronary angiography and were conscious and able to communicate, to understand the purpose of the study, and to give consent. Criteria for exclusion were patients taking antianxiety or sleep medications or who had an aroma-essential-oil contraindication.

The subjects were informed of the purpose of the experiment, consent was given, and voluntary participation and withdrawal was also explained. The subjects were given a small gift after participation.

### 2.3. Sample Size Calculation

The sample size for this study was calculated using *G* ∗ Power analysis. With a significance level (*α*) of 0.05, a statistical power (1 − *β*) of 0.80, and an effect size of 0.74, as calculated from previous studies [14], we calculated a need for 30 aromatherapy and 30 control subjects. After factoring in the failure rate, data were collected from a total of 66 subjects, and 8 patients who transferred rooms during the experiment were excluded; therefore, the data from 28 subjects in the aromatherapy group and 28 subjects in the control group were analysed. 

### 2.4. Interventions

The experimental treatment was a refrigerated oil blend of lavender (*Lavandula officinalis*), roman chamomile (*Chamomile roman*), and neroli (*Citrus aurantium*) at a ratio of 6 : 2 : 0.5 as prescribed by an aromatherapist. Lavender suppresses heart stimulation and lowers blood pressure; therefore, it is useful in the treatment of heart acceleration and high blood pressure. Chamomile has a calming effect and is effective in relieving anxiety and stress, and neroli has a calming effect and is effective in treating insomnia [9]. The method of application was the inhalation of essential oil that was dropped on aroma stones. In the aromatherapy group, two drops of the blend of lavender, roman chamomile, and neroli oils at a ratio of 6 : 2 : 0.5 were inhaled, through 10 deep breaths, before and after PCI, and the aroma stone was placed under the patient's pillow until the following morning. The control group received conventional nursing care.

### 2.5. Outcome Measures

In this study, state and trait anxiety were assessed using the Spielberger's State-Trait Anxiety Inventory-form Korean YZ (STAI-KYZ) [15]. State and trait anxiety are analysed with 20 questions, and a higher score signifies a higher level of anxiety. The coefficient of reliability of the STAI-KYZ calculated for state and trait anxiety was Cronbach's *α* = .92. In this study, the coefficient of reliability for state anxiety was Cronbach's *α* = .95 and for trait anxiety was Cronbach's *α* = .94. The Visual Analogue Scale (VAS) was used to measure the state anxiety periodically. VAS lets the subjects self-ascribe anxiety levels between 0 and 10 on a 10 cm horizontal line, with a higher score indicating a higher level of anxiety.

Blood pressure (BP) was measured using the Sure V24 patient monitor (Phillips, USA, 2004). The left brachium of the subject was raised to the heart level, the lower end of the cuff was strapped 2 cm anterior to the elbow, and measurements were taken according to the instrumental procedure. The BP was recorded in mmHg units. BP was measured once before PCI and 13 times after the procedure, for a total of 14 measurements.

Sleep quality was measured using the Korean translation form [16] of VSH Sleep Scale [17]. This tool consists of 8 questions about 4 sleep characteristics based on sleeping time, sleep disturbance, sleep onset, and sleep soundness, each scored on a 10-point scale, with a resulting total range between 0 point and 80 points. A higher score indicates more satisfied sleep, and the coefficient of reliability of the VSH sleep scale was Cronbach's *α* = .80 in their results [16]. In this study, the coefficient of reliability for preprocedure sleep was Cronbach's *α* = .86 and for postprocedure sleep was Cronbach's *α* = .94.

### 2.6. Data Collection

Demographic characteristics and baseline anxiety, BP, and sleep quality were assessed on the day of admission. Outcome measures including BP, anxiety, and sleep quality were assessed again before and after PCI. BP was measured every hour for 12 hours, for a total of 13 measurements. Outcomes as anxiety, BP, and sleep quality were assessed at 9AM on the day following PCI.

### 2.7. Data Analysis

The data were analysed with the PASW Statistic 18.0 program. The general characteristics of the subjects were analysed using raw numbers and percentages. The homogeneity tests of the general characteristics of the aromatherapy and control groups were performed using the *χ* ^2^-test and the *t*-test. Anxiety, sleep quality, and BP before and after the treatment in the aromatherapy and control groups were analyzed using the *t*-test and repeated-measures ANCOVA. The coefficients of reliability of the experimental tools were analysed with the Cronbach's *α*.

## 3. Results

### 3.1. Homogeneity Test of Subjects

There were no significant differences between the two groups in gender, age, marital status, level of education, exercise, smoking, alcohol consumption, state and trait anxiety, sleep quality, and BP. The homogeneity test of disease-related characteristics also indicated no significant differences between the two groups in terms of PCI experience, site of insertion, and ICU experience (Table 1).

### 3.2. Aromatherapy for the Anxiety and Sleep Quality

**Figure 1:**
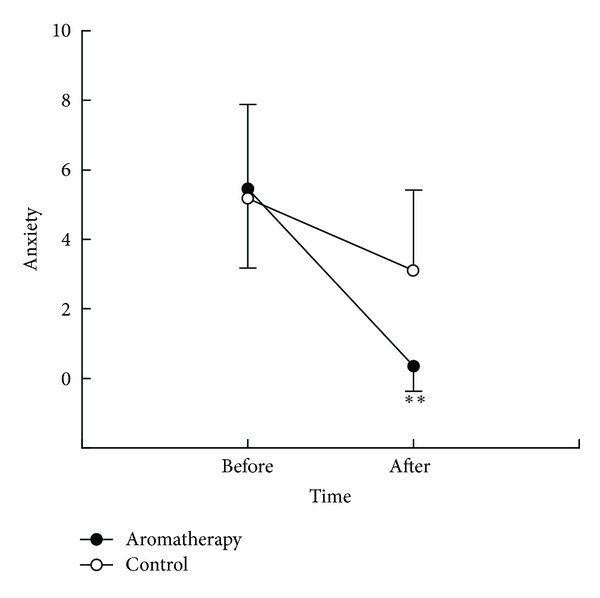
Pre- and postmeasurement of anxiety. **P < .01 between 2 groups. Data are expressed as mean and standard deviation.

After treatment, the anxiety levels were 0.36 (SD, 0.73) in the aromatherapy group and 3.11 (SD, 2.31) in the control group (*t* = 5.99, *P* < .001) (Figure 1). The changes in anxiety level were 5.10 (SD, 2.06) in the aromatherapy group and 2.07 (SD, 2.55) in the control group. There was a significant reduction in the aromatherapy group compare with the control group (*t* = −4.90, *P* < .001).

**Figure 2:**
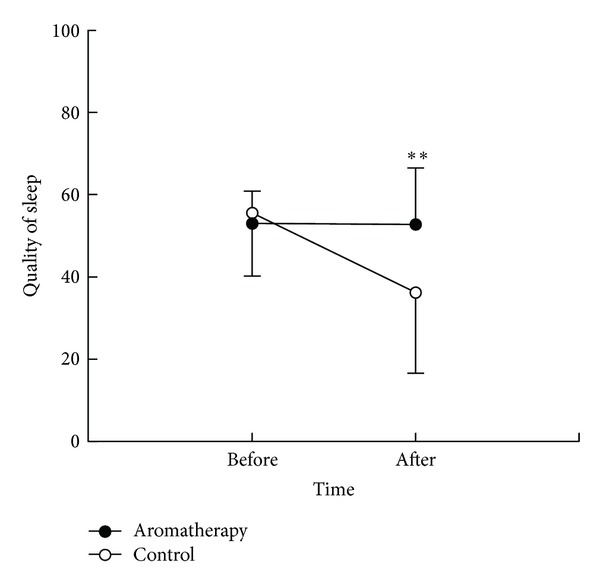
Pre- and postmeasurement of quality of sleep. **P < .01 between 2 groups. Data are expressed as mean and standard deviation.

After the experimental treatment, the aromatherapy group had a sleep score of 52.7 (SD, 13.8), whereas the control group had a score of 36.2 (SD, 19.6), showing a significant difference between the two groups (*t* = −3.65, *P* = .001) (Figure 2). There was a significant difference in changes of sleep score between the aromatherapy group (0.28, SD, 17.13) and the control group (19.39, SD, 17.30) (*t* = 4.15, *P* < .001).

### 3.3. Aromatherapy for the BP

**Figure 3:**
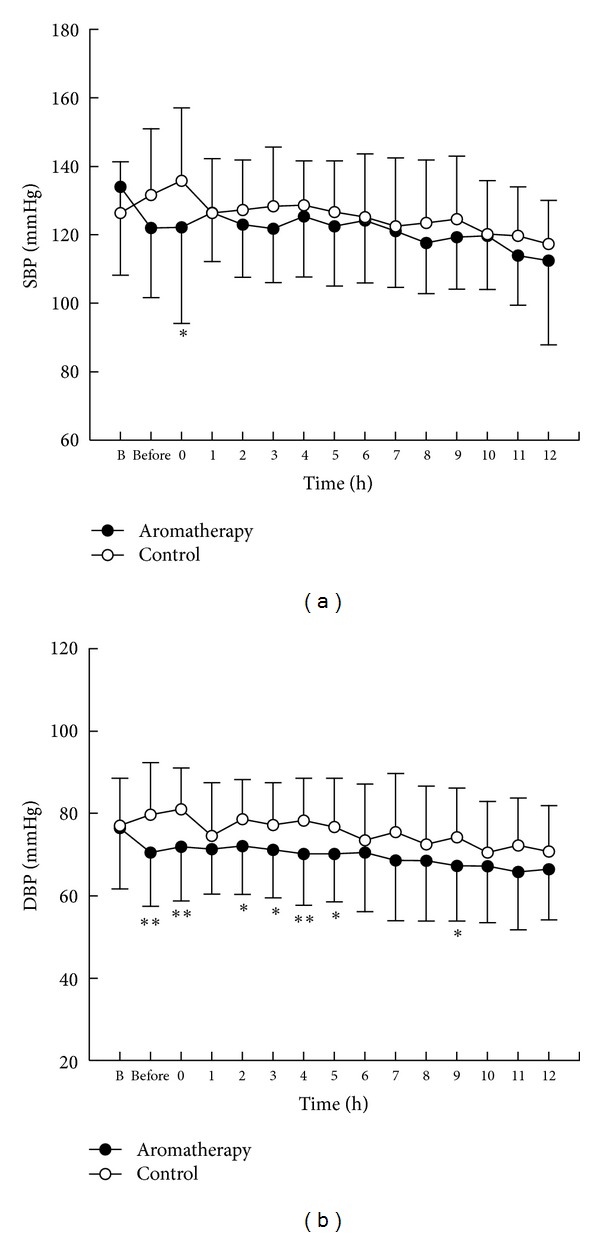
Changes of (a) systolic blood pressure (SBP) and (b) diastolic blood pressure (DBP) in aromatherapy and control groups. *P < .05 and **P < .01 between 2 groups. Data are expressed as mean and standard deviation. The ANCOVA test showed the main effects of time (*F* = 0.85, *P* = 0.60), group (*F* = 4.63, *P* = 0.036), and group × time interaction (*F* = 1.29, *P* = 0.32) in (a) SBP and time (*F* = 1.33, *P* = 0.24), group (*F* = 6.93, *P* = 0.011), and group × time interaction (*F* = 0.64, *P* = 0.81) in (b) DBP. PCI: percutaneous coronary intervention.

The systolic blood pressure (SBP) and diastolic blood pressure (DBP) on the day of admittance were used as the covariates for analysis. There were neither significant differences based on time nor any interaction between time and group; however, there was a significant difference between the groups in both SBP (*F* = 4.63, *P* = .036) and DBP (*F* = 6.93, *P* = .011) (Figures 3(a) and 3(b)).

## 4. Discussion

This study was a nonequivalent control group nonsynchronised assessment of the effect of aromatherapy on the anxiety, sleep, and BP of cardiac stent insertion patients admitted to the cardiovascular ICU. The aromatherapy had a positive effect on reducing anxiety, increasing sleep, and stabilising BP in the patients in the cardiovascular ICU after cardiac stent insertion; therefore, it may be used as an independent nursing intervention.

The anxiety level of the aromatherapy group was significantly lower than that of the control group. These results are consistent with the anxiety-decreasing effects of aromatherapy in patients before surgery [18], during menstruation [19], in haemodialysis patients [12], and during colonoscopy [13].

After treatment, the aromatherapy group experienced no significant changes in sleep quality, whereas the control group had significantly worse sleep. Assuming that sleep is generally decreased in patients in the ICU and because the aromatherapy group did not have a decrease in sleep, we can conclude that aromatherapy is effective in maintaining sleep despite a stressful situation. This result is consistent with those of previous studies of increased sleep satisfaction among nightshift nurses [20], haemodialysis patients [12], and coronary angiography patients [14] upon aromatherapy application.

There were neither a significant difference in BP based on time nor any interaction between time and group; however, there was a significant difference between the groups in both SBP and DBP. Other authors have reported various results, including a decrease in BP [10] and no effect on BP [18, 21]. In the positive study [10], lavender, ylang-ylang, and bergamot oils were used, whereas in the negative studies, lavender and bergamot [21] or lavender, ylang-ylang, and bergamot [18] were used. In this study, we used lavender, roman chamomile, and neroli oils. A comparison of the results of this study with those of the previous studies indicates that the type of oil and its properties affect BP results. The essential aroma oils used in this study have calming and BP-lowering effects; therefore, they may have caused the decrease of SBP and DBP. However, because the changes in BP did not deviate significantly from normal BP ranges, further research is necessary to determine the clinical utility of this effect. It is noteworthy that the BP in the control group increased immediately before and after the PCI procedure in comparison to the day of admission, whereas, in the aromatherapy group, the BP after aromatherapy treatment and before the PCI procedure was approximately 12 mmHg lower than on the day of admission, and the BP was maintained at a similar level. Therefore, aromatherapy may negate the BP-raising effects of stress, although more rigorous research on this topic is necessary.

During this study, there were no reports of headache or nausea associated with the administration of aromatherapy. Most of the subjects reported that the aromatherapy inhalation had a pleasant odour. They also indicated their satisfaction in being treated in a foreign environment such as an ICU after the procedure.

In summary, aromatherapy reduced anxiety, increased sleep, and stabilised the BP of patients undergoing cardiac stent insertion. Among alternative therapies that have recently been introduced, aromatherapy is easy to apply, fast-acting and can be used in independent nursing interventions. More research is necessary for it to become a suitable nursing intervention in practice.

## Figures and Tables

**Table 1:** Homogeneity test for general characteristics.

Categories	Aromatherapy group (*n* = 28)	Control group (*n* = 28)	*χ* ^2^	*P*
	*n* (%)	*n* (%)		
Gender				
Male	18 (64.3)	24 (85.7)	3.43	.12*
Female	10 (35.7)	4 (14.3)		
Age (yr)				
≤60	8 (28.6)	15 (53.6)	3.89	.14
61–70	12 (42.8)	9 (32.1)		
≥71	8 (28.6)	4 (14.3)		
Marital status				
Unmarried	0 (0)	1 (3.6)	1.02	1.0*
Married	28 (100)	27 (96.4)		
Education				
Lower than middle school	21 (75.0)	13 (46.4)		
High school	6 (21.4)	10 (35.7)	7.44	.11
More than college	1 (3.6)	5 (17.9)		
Exercise				
Yes	6 (21.4)	14 (50.0)	8.06	.09
No	22 (78.6)	14 (50.0)		
Amount of smoking (cigarettes per day)				
None	20 (71.5)	18 (64.3)	4.08	.25
≤10	3 (10.7)	2 (7.1)		
≥10, ≤20	3 (10.7)	1 (3.6)		
≥20	2 (7.1)	7 (25.0)		
Alcohol consumption				
No	20 (71.4)	18 (64.3)	0.33	.57
Yes	8 (28.6)	10 (35.7)		
Prior PCI				
Yes	17 (60.7)	17 (60.7)	.00	1.0
No	11 (39.3)	11 (39.3)		
Site of Insertion				
Wrist site	13 (46.4)	15 (53.6)	.29	.79
Femoral site	15 (53.6)	13 (46.4)		
Prior ICU admission				
Yes	11 (39.3)	11 (39.3)	<.001	1.0
No	17 (60.7)	17 (60.7)		

	Mean ± SD	Mean ± SD	*t*	*P *

State anxiety	48.5 ± 8.4	44.8 ± 11.27	−1.37	.17
Trait anxiety	36.8 ± 6.1	41.6 ± 11.1	2.0	.05
Baseline Anxiety (VAS)	5.5 ± 2.3	5.2 ± 2.7	−0.43	.67
Quality of Sleep	53.0 ± 7.8	55.6 ± 15.4	0.79	.44
SBP	134.0 ± 25.8	126.5 ± 15.0	−1.34	.86
DBP	76.5 ± 14.9	77.2 ± 11.4	0.18	.86

*: Fisher's exact test; PCI: Percutaneous coronary intervention; ICU: Intensive Care Unit; Mean ± SD: Mean ± Standard Deviation; SBP: Systolic Blood Pressure; DBP: Diastolic Blood Pressure.
